# High-Resolution Inhibition Profiling Combined with HPLC-HRMS-SPE-NMR for Identification of PTP1B Inhibitors from Vietnamese Plants

**DOI:** 10.3390/molecules22071228

**Published:** 2017-07-20

**Authors:** Binh Thi Dieu Trinh, Anna K. Jäger, Dan Staerk

**Affiliations:** Department of Drug Design and Pharmacology, Faculty of Health and Medical Sciences, University of Copenhagen, Universitetsparken 2, DK-2100 Copenhagen, Denmark; binh.trinh@sund.ku.dk (B.T.D.T.); ds@sund.ku.dk (D.S.)

**Keywords:** type 2 diabetes, PTP1B, Vietnamese medicinal plants, *Ficus racemosa*, isoflavonoids

## Abstract

Protein tyrosine phosphatase 1B (PTP1B) plays a key role as a negative regulator in insulin signal transduction by deactivating the insulin receptor. Thus, PTP1B inhibition has emerged as a potential therapeutic strategy for curing insulin resistance. In this study, 40 extracts from 18 different plant species were investigated for PTP1B inhibitory activity in vitro. The most promising one, the EtOAc extract of *Ficus racemosa*, was investigated by high-resolution PTP1B inhibition profiling combined with HPLC-HRMS-SPE-NMR analysis. This led to the identification of isoderrone (**1**), derrone (**2**), alpinumisoflavone (**3**) and mucusisoflavone B (**4**) as PTP1B inhibitors. IC_50_ of these compounds were 22.7 ± 1.7, 12.6 ± 1.6, 21.2 ± 3.8 and 2.5 ± 0.2 µM, respectively. Kinetics analysis revealed that these compounds inhibited PTP1B non-competitively with *K*_i_ values of 21.3 ± 2.8, 7.9 ± 1.9, 14.3 ± 2.0, and 3.0 ± 0.5 µM, respectively. These findings support the important role of *F. racemosa* as a novel source of new drugs and/or as a herbal remedy for treatment of type 2 diabetes.

## 1. Introduction

Diabetes mellitus has become a major and growing public health problem worldwide, affecting 415 million people and causing 5 million deaths in 2015. The number of people with diagnosed diabetes is expected to increase by 55% between 2015 and 2040 to reach 642 million people, with the majority of cases in low- and middle-income countries [1]. Type 2 diabetes (T2D), also known as noninsulin-dependent diabetes, is the predominant form and causes more than 90% of all diabetes cases [2]. The insulin resistance observed in T2D patients is caused by the inability of the cells to propagate the insulin signal [3]. This results in postprandial hyperglycemia, causing severe long-term complications, such as diabetic neuropathy, diabetic retinopathy, diabetic nephropathy and cardiovascular diseases [4,5,6]. The insulin-signaling pathway is one of the key pathways responsible for blood glucose regulation. When binding to its receptor, insulin triggers the autophosphorylation of the insulin receptor and insulin receptor substrates (IRS). This activates several signaling cascades, leading to biological responses such as glucose uptake into the cell and glycogen synthesis. Protein-tyrosine phosphatase 1B (PTP1B) is an intracellular enzyme found in insulin-targeted tissues such as liver, muscle, and fat. Acting as a negative regulator in the insulin-signaling pathway, PTP1B can dephosphorylate, and thereby, deactivate the insulin receptor. Inhibition of PTP1B can reduce the resistant state. This has been validated in a PTP1B knockout mice experiment, where insulin sensitivity was enhanced in muscle and liver. On a high-fat diet, these mice were also resistant to weight gain and remained insulin sensitive [7]. Therefore, PTP1B has become a promising therapeutic target for the treatment of type 2 diabetes [7,8,9]. A large number of PTP1B inhibitors discovered from both natural and synthetic origin have been developed and investigated for their ability to stimulate insulin signaling. However, none of them are approved for clinical use due to the poor cell permeability and low bioavailability, which limit their potential as effective drugs [10].

For many years, natural products have been considered a valuable source of effective antihyperglycemic phytochemicals. Several prenylated flavonoids, coumarins, terpenoids and related compounds with affinity towards PTP1B have been identified [11]. Due to the chemical complexity of plant extracts, identification of bioactive compounds as well as their isolation and structure elucidation are obviously a difficult and time-consuming task. Simultaneously, minor components with interesting bioactivity may be missed because their concentrations are below the detection threshold of the biological screening or masked by other compounds in the complex mixture. In this study, we therefore used high-resolution inhibition profiling, in which 96-well microfractionation of eluate from analytical-scale high-performance liquid chromatography is followed by bioassaying of the material in each well. This provides a high-resolution inhibition profile that can be overlaid with the corresponding HPLC chromatogram to allow direct pinpointing of HPLC peaks correlated with bioactivity. These peaks were subsequently analyzed using hyphenated high-performance liquid chromatography, high-resolution mass spectrometry, solid-phase extraction, and nuclear magnetic resonance, i.e., HPLC-HRMS-SPE-NMR, which allows targeted isolation and structural characterization of bioactive compounds directly from crude extract. The combined HR-bioassay/HPLC-HRMS-SPE-NMR platform has been successfully applied for identification of bioactive constituents from natural sources such as plant and food extract [12,13,14,15,16,17]. Here, we report crude extract screening of 40 Vietnamese plant extracts (from 18 different plant species used for the treatment of type 2 diabetes) for PTP1B inhibitors, followed by high-resolution PTP1B inhibition profiling in combination with HPLC-HRMS-SPE-NMR analysis for identification of bioactive constituents.

## 2. Results and Discussion

### 2.1. PTP1B Inhibitory Activity of Crude Extracts

**Figure 1:**
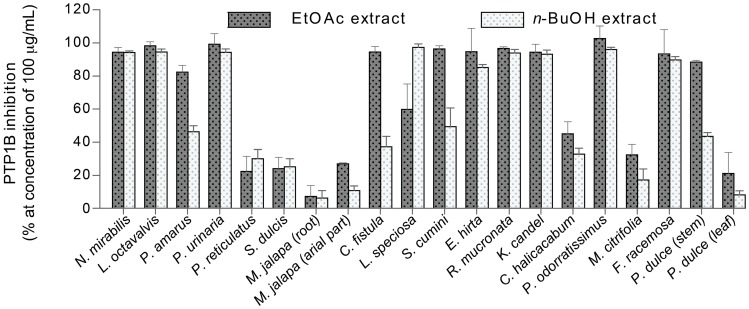
Protein tyrosine phosphatase 1B (PTP1B) inhibitory activity of EtOAc and *n*-BuOH extracts of 18 different plant species, at a concentration of 100 µg/mL, represented as mean ± standard deviation, *n* = 3.

A total of 40 extracts from 18 different plant species (Table 1) were investigated. The EtOAc and *n*-BuOH extracts were tested for their ability to inhibit PTP1B at a concentration of 100 µg/mL, and extracts showing more than 80% inhibition (Figure 1) were considered to be active. 

Thus, the EtOAc extracts of *N. mirabilis*, *L. octovalvis*, *P. amarus*, *P. urinaria*, *C. fistula*, *S. cumini*, *E. hirta*, *R. mucronata*, *K. candel*, *P. odoratissimu*, *F. racemosa*, and *P. dulce*, and the *n*-BuOH extracts of *N. mirabilis*, *L. octovalvis*, *P. urinaria*, *L. speciosa*, *E. hirta*, *R. mucronata*, *K. candel*, *P. odoratissimus*, and *F. racemosa* were selected for IC_50_ value determination. Crude extracts were dissolved in DMSO to obtain a stock concentration of 10 mg/mL and serial ten-fold dilutions were performed six times. Results showed that all these extracts strongly inhibited PTP1B with IC_50_ values ranging from 0.02 to 74.4 µg/mL (IC_50_ of RK682 = 3.8 ± 0.6 µg/mL). The IC_50_ values of active extracts were calculated from the dose-response curves (Supplementary data, Figures S1 and S2) and summarized in Table 1. Previously, PTP1B inhibitory activity has been reported for a MeOH extract of *P. reticulatus* (IC_50_ = 4.5 µg/mL [18]) and an EtOH extract of *F. racemosa* (IC_50_ = 12.1 µg/mL [19]). Besides this, EtOAc and MeOH extracts of *S. cumini* have been reported as strong PTP1B inhibitors (IC_50_ = 1.9 ± 0.1 and 38.2 ± 0.1 µg/mL, respectively [20,21]). From the EtOAc extract, jamunones A–H, jamunones J–K, jamunones M–O and spiralisone C were revealed as potent PTP1B inhibitors with IC_50_ values ranging from 0.42 to 3.2 µM.

### 2.2. High-Resolution PTP1B Inhibition Profiles

**Figure 2:**
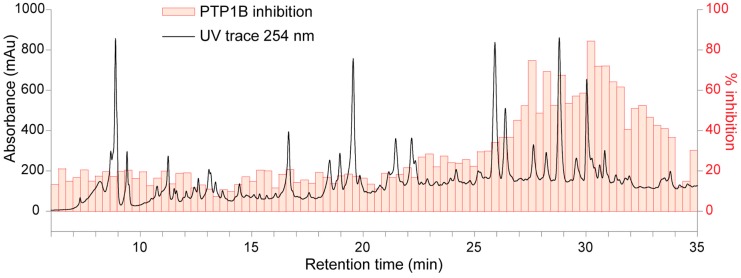
HPLC trace at 254 nm and high-resolution PTP1B inhibition profile of EtOAc extract of *Ficus racemosa*.

All the active extracts were subjected to an analytical-scale HPLC (10 µL of 30 mg/mL extract), using separation condition as described in Section 3.4. The chromatograms showed that most extracts contained large amounts of tannins based on the large hump from 7 to 22 min (Supplementary data, Figures S3 and S4). Tannins are polyphenolic compounds widely distributed in many plant species, and are generally considered as non-specific inhibitors, which have low priority for drug discovery. Based on the HPLC chromatograms, only EtOAc extracts of *P. amarus*, *C. fistula*, *S. cumini*, *E. hirta*, *F. racemosa* and *n*-BuOH extracts of *L. speciosa* and *F. racemosa* had low levels of tannins. Chromatographic separation of these extracts was thus optimized (described in Section 3.5) before being subjected to a time-based microfractionation in 96-well microplates, followed by evaporation of the HPLC eluates and assessment of the PTP1B inhibitory activity of all wells. The inhibitory activities (calculated as percentage inhibition) were plotted against the retention time from the microfractionation to give the high-resolution PTP1B inhibition profile (biochromatogram). The HPLC chromatogram at 254 nm is shown with the black line and the high-resolution PTP1B inhibition profile shown with the red bars (each bar represents inhibition of eluates in a well). The high-resolution PTP1B inhibition profiles of EtOAc extracts of *P. amarus*, *C. fistula*, *S. cumini*, and *E. hirta* and *n*-BuOH extracts of *L. speciosa* and *F. racemosa* (Supplementary data, Figure S5) showed no distinct peaks with PTP1B inhibition despite the significant inhibitory activities in the crude extracts. This can be caused by loss of synergistic activities of constituents that are separated by microfractionation, but assessed collectively by the crude extract screening. Therefore, no further investigation of these extracts was performed. An inhibition region was observed from 27 to 34 min in the biochromatogram of *F. racemosa* EtOAc extract which correlated to highly UV-absorbing signals (Figure 2). Because the biochromatogram could not pinpoint clearly the active compounds, the *F. racemosa* EtOAc extract was subjected to preparative-scale HPLC to obtain a fraction containing these signals (namely Fr.1, 12 mg) for further analysis.

### 2.3. HPLC-HRMS-SPE-NMR Analysis

**Figure 3:**
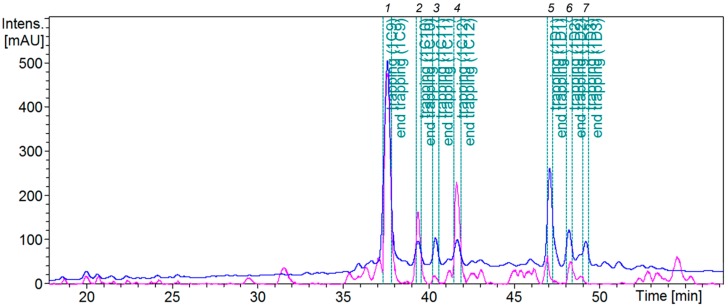
Trapping peaks *1*–7 of Fr.1 of *Ficus racemosa* EtOAc extract, analyzed by HPLC-PDA-HRMS-SPE-NMR using an analytical-scale HPLC. Blue line: UV chromatogram at 254 nm, pink line: base peak chromatogram.

The HPLC elution profile of Fr.1 was re-optimized to improve separation of active peaks. UV thresholds-based SPE trapping was set up to collect peaks *1*–*7* (Figure 3), using separation condition as described in Section 3.7.

**Figure 4:**
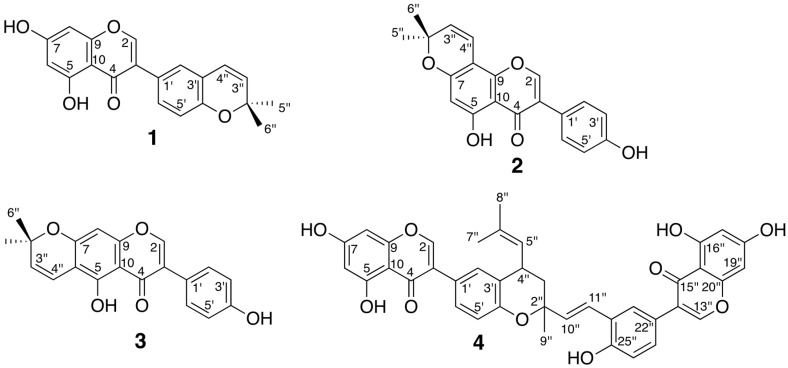
Structures of compounds **1**–**4** isolated from EtOAc extract of *Ficus racemosa*.

On the basis of HRMS as well as 1D NMR spectra obtained from HPLC-HRMS-SPE-NMR analysis, peaks *1*, *2* and *4* were assigned to isoderrone (**1**), C_20_H_16_O_5_, HR-ESIMS(+) *m*/*z* 337.1083 [M + H]^+^, ^1^H-NMR (600 MHz, acetonitrile-*d*_3_) ppm: δ 12.91 (1H, s, OH-5), 8.02 (1H, s, H-2), 7.29 (1H, dd, *J* = 8.3, 2.2 Hz, H-6′), 7.22 (1H, d, *J* = 2.2 Hz, H-2′), 6.79 (1H, d, *J* = 8.3 Hz, H-5′), 6.42 (1H, d, *J* = 9.8 Hz, H-4′′), 6.40 (1H, d, *J* = 2.2 Hz, H-8), 6.27 (1H, d, *J* = 2.2 Hz, H-6), 5.75 (1H, d, *J* = 9.8 Hz, H-3′′), 1.42 (6H, s, H_3_-5′′, H_3_-6′′) [22]; derrone (**2**), C_20_H_16_O_5_, HR-ESIMS(+) *m*/*z* 337.1074 [M + H]^+^, ^1^H-NMR (600 MHz, acetonitrile-*d*_3_) ppm: δ 13.02 (1H, s, OH-5), 8.07 (1H, s, H-2), 7.40 (2H, d, *J* = 8.6 Hz, H-2′, H-6′), 6.89 (2H, d, *J* = 8.6 Hz, H-3′, H-5′), 6.72 (1H, d, *J* = 10.0 Hz, H-4′′), 6.22 (1H, s, H-6), 5.71 (1H, d, *J* = 10.0 Hz, H-3′′), 1.46 (6H, s, H_3_-5′′, H_3_-6′′) [23]; alpinumisoflavone (**3**), C_20_H_16_O_5_, HR-ESIMS(+) *m*/*z* 337.1073 [M + H]^+^, ^1^H-NMR (600 MHz, acetonitrile-*d*_3_) ppm: δ 13.33 (1H, s, OH-5), 8.01 (1H, s, H-2), 7.39 (2H, d, *J* = 8.6 Hz, H-2′, H-6′), 6.88 (2H, d, *J* = 8.6 Hz, H-3′, H-5′), 6.68 (1H, d, *J* = 10.0 Hz, H-4′′), 6.37 (1H, s, H-8), 5.74 (1H, d, *J* = 10.0 Hz, H-3′′), 1.45 (6H, s, H_3_-5′′, H_3_-6′′) [24]. The structures of compound **1**, **2**, **3** are shown in Figure 4. The low content of the metabolites in the extract did not allow the trapping of peaks *3*, *5*, *6* and *7* with a quantity and purity sufficient for NMR experiments.

### 2.4. Isolation and Evaluation of Bioactive Compounds 

From 40.0 g dried material of *F. racemosa*, 450 mg EtOAc extract was obtained and subsequently subjected to preparative-scale HPLC (described in Section 3.6) to obtain the material eluted as peak *1* (3.5 mg), *2* (1.2 mg), *3* (0.7 mg), *4* (1.5 mg), *5* (1.2 mg), *6* (0.6 mg), and *7* (0.4 mg). On the basis of HRMS as well as 1D and 2D NMR spectra, peak *5* was assigned to mucusisoflavone B (**4**), C_40_H_32_O_10_, HR-ESIMS(+) *m*/*z* 673.2068 [M + H]^+^, ^1^H-NMR (600 MHz, acetone-*d*_6_) ppm: δ 13.03 (2H, s, OH-5, OH-16′′), 8.24 (1H, s, H-13′′), 8.14 (1H, s, H-2), 7.74 (1H, d, *J* = 2.1 Hz, H-23′′), 7.37 (1H, dd, *J* = 8.3, 2.1 Hz, H-27′′), 7.35 (1H, dd, *J* = 8.2, 2.0 Hz, H-6′), 7.33 (1H, d, *J* = 2.0 Hz, H-2′), 7.10 (1H, d, *J* = 16.2 Hz, H-11′′), 6.96 (1H, d, *J* = 8.3 Hz, H-26′′), 6.90 (1H, d, *J* = 8.2 Hz, H-5′), 6.60 (1H, d, *J* = 16.2 Hz, H-10′′), 6.43 (1H, d, *J* = 2.2 Hz, H-19′′), 6.42 (1H, d, *J* = 2.2 Hz, H-8), 6.29 (1H, d, *J* = 2.2 Hz, H-17′′), 6.28 (1H, d, *J* = 2.2 Hz, H-6), 5.19 (1H, m, H-5′′), 3.92 (1H, m, H-4′′), 2.12 and 1.80 (each 1H, overlapping signals, H_2_-3′′), 1.84 (3H, s, H_3_-7′′), 1.78 (3H, s, H_3_-8′′), 1.55 (3H, s, H_3_-9′′); ^13^C-NMR (150 MHz, acetone-*d*_6_) ppm: δ 181.6 (C-4, C-15′′), 165.2 (C-7, C-18′′), 163.9 (C-5, C-16′′), 159.1 (C-9), 159.0 (C-20′′), 155.7 (C-4′, C-25′′), 154.6 (C-2), 154.3 (C-13′′), 136.3 (C-10′′), 134.0 (C-6′′), 130.6 (C-2′), 130.2 (C-27′′), 129.3 (C-6′), 128.5 (C-23′′), 128.3 (C-5′′), 125.4 (C-3′), 124.8 (C-22′′), 124.2 (C-3), 124.0 (C-14′′), 123.6 (C-1′), 123.5 (C-24′′), 123.2 (C-11′′), 117.9 (C-5′), 116.5 (C-26′′), 106.1 (C-10, C-21′′), 99.9 (C-6, C-17′′), 94.5 (C-8, C-19′′), 77.4 (C-2′′), 40.1 (C-3′′), 32.6 (C-4′′), 26.0 (C-8′′), 24.4 (C-9′′), 18.1 (C-7′′) [25]. Peaks *3*, *6* and *7* were impure and the subsequent purification of these peaks was not successful despite several efforts. No further attempt was made to identify these three PTP1B inhibitors due to the limited amount of *F. racemosa* available to us. 

**Figure 5:**
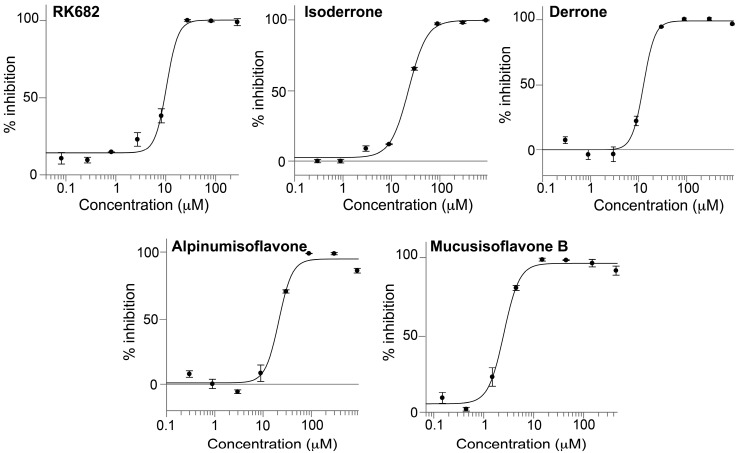
Dose–response curves of compounds **1**–**4**. Each point represents the average of triplicate measurements.

All isolated compounds were accessed for in vitro PTP1B inhibitory activity with RK682 as reference compound. Compounds were dissolved in DMSO and dilution series were prepared for IC_50_ determination. The dose-response curves are shown in Figure 5 and the IC_50_ values of all tested compounds are given in Table 2. These results showed that mucusisoflavone B (**4**), an isoflavone dimer derivative, significantly inhibited PTP1B with an IC_50_ value of 2.5 ± 0.2 µM, which is four times lower than that of RK682 (10.4 ± 1.6 µM). The other isoflavonoids also displayed a strong inhibition of PTP1B with IC_50_ values of 22.7 ± 1.7 µM (isoderrone, **1**), 12.6 ± 1.6 µM (derrone, **2**), and 21.2 ± 3.8 µM (alpinumisoflavone, **3**). The comparison of compounds **1**–**3** showed that the PTP1B inhibitory activity was in order of **2** > **3** ~ **1**, suggesting that the presence of a 2,2-dimethylpyran ring system at C-7 and C-8 produced higher inhibitory activity of compound **2**. The 2,2-dimethylpyran ring system located at C-3′ and C-4′ or at C-6 and C-7, as observed in compounds **1** or **3**, might be responsible for the decrease in inhibitory activity. Compounds **1**–**3** have previously been reported to possess PTP1B inhibitory activity. While compound **1** inhibited PTP1B 78% at a concentration of 25 µM [26], IC_50_ of compounds **2** and **3** against PTP1B were 20.63 ± 0.17 µM and 37.52 ± 0.31 µM, respectively [27]. Thus, our results are in agreement with the previous reports. No previous investigation of the PTP1B inhibitory activity of mucusisoflavone B (**4**) has been reported.

### 2.5. Kinetics of PTP1B Inhibition

**Figure 6:**
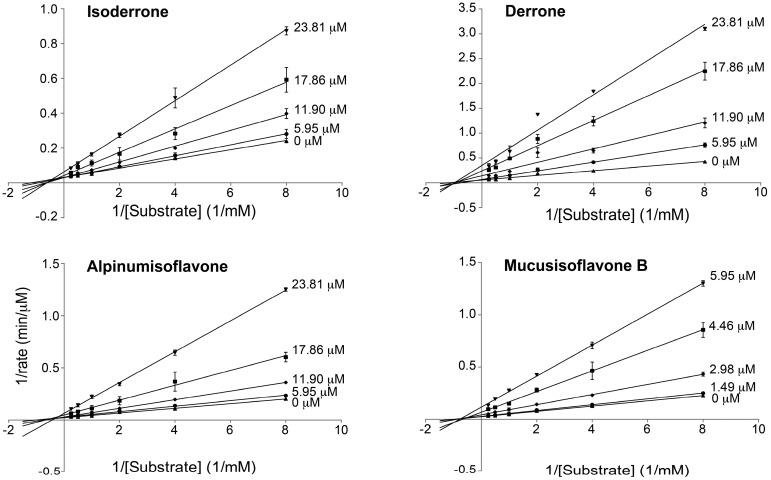
Lineweaver–Burk plots of inhibition kinetics of PTP1B inhibitory effects by compounds **1**–**4**. Each point represents the average of triplicate measurements.

In order to examine the mode of inhibition of PTP1B by compounds **1**–**4**, kinetic analyses were carried out. As shown in Figure 6, all compounds inhibited in a noncompetitive manner, as increasing the concentration of inhibitors reduced the maximum rate of the enzymatic reaction (*V*_max_) without changing the binding affinity of the enzyme for the substrate (*K*_m_). During this mode of inhibition, the substrate and the inhibitor simultaneously bind to the enzyme at different sites and the ESI complex cannot form the product. *K*_i_ is the most important measurement for an inhibitor in noncompetitive inhibition, as it is an indication of the affinity for an enzyme. *K*_i_ values of compounds **1**–**4** were calculated to be 21.3 ± 2.8 µM, 7.9 ± 1.9 µM, 14.3 ± 2.0 µM, 3.0 ± 0.5 µM, respectively, suggesting that compound **4** is the most tightly bound to the enzyme. Though IC_50_ values of compounds **1** and **3** are comparatively equal, the *K*_i_ value of compound **3** is lower than that of compound **1**, indicating that compound **3** is a more potent inhibitor. The *K*_i_ values and mode of inhibition for compounds **1**–**4** are summarized in Table 2. This is the first report on the kinetics of PTP1B inhibition of these compounds. 

## 3. Materials and Methods

### 3.1. Reagents

CDCN_3_, methanol-*d*_4_, dimethyl sulfoxide (DMSO), *p*-nitrophenyl phosphate (*p*NPP), tris-(hydroxymethyl)aminomethane (Tris), bis-(2-hydroxyethyl)amino-tris-(hydroxymethyl) methane (Bis-Tris), dl-dithiothreitol (DTT), ethylenediaminetetraacetatic acid (EDTA), RK682 were purchased from Sigma-Aldrich (St. Louis, MO, USA). Formic acid was obtained from Merck (Darmstadt, Germany). Recombinant human protein tyrosine phosphatase 1B (PTP1B) (BML-SE332-0050, EC 3. 1. 3. 48) was purchased from Enzo Life Sciences Inc., (Farmingdale, NY, USA). All solvents used were HPLC grade, and water was purified by a deionization and 0.22-µm membrane filtration system (Milipore, Billerica, MA, USA).

### 3.2. Plant Material and Sample Preparation

Samples were collected from the Southern part of Vietnam in June 2014, and identified by Son V. Dang, Curator of the VNM Herbarium, Institute of Tropical Biology, Vietnam. Voucher specimens are stored at the General Herbarium of Vascular Plants, National History Museum, University of Copenhagen. All the plant species, the parts used and voucher numbers are presented in Table 1. The dried and coarsely powdered plant material (2 g) was sonicated in 20 mL MeOH at room temperature for two hours and left overnight. The filtrate was evaporated under reduced pressure to yield a dry residue which was then suspended in H_2_O (20 mL) and defatted with *n*-hexane (3 × 20 mL). The hexane layer was discarded while the water layer was partitioned successively with EtOAc (3 × 20 mL) and *n*-BuOH (3 × 20 mL) to produce EtOAc and *n*-BuOH extracts. 

### 3.3. In Vitro PTP1B Inhibition Assay

Based on the method described by Tahtah et al. [28], the PTP1B inhibition assay was performed at 25 °C using a two-component buffer, containing 50 mM Tris, 50 mM bis-Tris and 100 mM NaCl, adjusted to pH 7.0 with acetic acid. The final reaction volume was 180 µL, including 18 µL test sample dissolved in DMSO, 52 µL of buffer with 3.46 mM EDTA (final well concentrations 10% DMSO and 1 mM EDTA), and 60 µL of buffer with 1.5 mM *p*NPP and 6 mM DTT (final well concentrations 0.5 mM *p*NPP and 2 mM DTT). After preincubation at 25 °C for 10 min, the reaction was started by addition of 50 µL of 0.001 µg/µL PTP1B stock solution (final well concentration: 0.05 µg/well). The hydrolysis rate of *p*NPP to release *p*-nitrophenol was determined by measuring the absorbance at 405 nm every 30 s for 10 min. Preincubation and absorbance measurements were performed with a Multiscan FC microplate photometer with a built-in incubator (Thermo Scientific, Waltham, MA, USA) coupled to SkanIt version 2.5.1 software for data acquisition. The PTP1B inhibitory activity was expressed as percentage inhibition and was calculated using the following formula:

% inhibition = (Slope_blank_ − Slope_sample_)/Slope_blank_ × 100

RK682 was used as a positive control and all measurements were performed in triplicate. Dose-response curves and IC_50_ values were obtained using GraFit version 5 (Erithacus Software Ltd., Horley, UK).

### 3.4. Analytical-Scale HPLC Separation

Active extracts were dissolved in methanol and filtered using a Nylon Target Syringe Filter (0.45 µm pore size). Chromatographic separation was performed with an Agilent 1200 series instrument (Santa Clara, CA, USA) consisting of a quaternary pump, a degasser, a thermostatted column compartment, a photodiode-array detector, a high-performance auto sampler, and a fraction collector, all controlled by Agilent ChemStation ver. B.03.02 software. The column used was Luna C_18_(2) (Phenomenex, 150 × 4.6 µm, 3 µm particle size, 100 Å pore size) maintained at 40 °C. HPLC solvent A consisted of water–acetonitrile 95:5 and solvent B consisted of acetonitrile–water 95:5, both acidified with 0.1% formic acid. The flow rate was maintained at 0.5 mL/min with the following standard gradient: 0 min, 0% B; 30 min, 100% B; 35 min, 100% B; 36 min, 0% B and 5 min of equilibration. UV traces were monitored at 210, 235, 254, 280, 330 and 440 nm.

### 3.5. High-Resolution PTP1B Inhibition Profiling

Microfractionation of selected active extracts was performed on the same instrument, with the same conditions as described above. For a single injection (10 µL of a 30 mg/mL extract), eluates from 1 to 30 min (EtOAc extract of *P. amarus* and *C. fistula*, *n*-BuOH extract of *L. speciosa*) and from 6 to 35 min (EtOAc extract of *F. racemosa*) were fractionated into 88 wells of a 96-well microplate, while eluates from 7 to 23 min (EtOAc extract of *S. cumini* and *E. hirta*) and from 3 to 19 min (*n*-BuOH extract of *F. racemosa*) were fractionated into 48 wells of a 96-well microplate. This leads to a resolution of 3.0 data points per min. The following HPLC gradients were used: gradient I for separation of EtOAc extract of *P. amarus*: 0 min, 0% B; 1 min, 10% B; 20 min, 35% B; 26 min, 45%; 31 min, 100% B; 36 min, 100% B; 37 min, 0% B; and 5 min of equilibration; gradient II for separation of EtOAc extract of *E. hirta* and *S. cumini*: 0 min, 0% B; 1 min, 10% B; 20 min, 30% B; 30 min, 80% B; 31 min, 100% B; 36 min, 100% B; 37 min, 0% B; and 5 min of equilibration; standard gradient for separation of EtOAc extract of *F. racemosa*: 0 min, 0% B; 30 min, 100% B; 35 min, 100% B; 36 min, 0% B; and 5 min of equilibration; gradient III for separation of EtOAc extract of *C. fistula*: 0 min, 0% B; 1 min, 10% B; 30 min, 100% B; 35 min, 100% B; 36 min, 0% B; and 5 min of equilibration; gradient IV for separation of *n*-BuOH extract of *L. speciosa*: 0 min, 0% B; 1 min, 10% B; 20 min, 50% B; 25 min, 90% B; 30 min, 100% B; 35 min, 100% B; 36 min, 0% B; and 5 min of equilibration; gradient V for separation of *n*-BuOH extract of *F. racemosa*: 0 min, 0% B; 1 min, 5% B; 20 min, 20% B; 30 min, 100% B; 35 min, 100% B; 36 min, 0% B; and 5 min of equilibration. The microplates were subsequently evaporated to dryness using a Savant SPD121P speed vacuum concentrator. The PTP1B inhibition activities for each well were determined as described above, but replacing DMSO of test samples by DMSO for dissolution of fractionated material. For each microplate, a positive control (RK682) as well as a negative control (blank) was included in triplicate. High-resolution inhibition profiles were plotted by representing PTP1B inhibitory activity against chromatographic retention time.

### 3.6. Preparative-Scale HPLC Separation

The preparative-scale HPLC was performed with an Agilent 1100 series instrument (Santa Clara, CA, USA) consisting of a quaternary pump, a degasser, a thermostated column compartment, a photodiode-array detector, a high-performance auto sampler, and a fraction collector, all controlled by Agilent ChemStation ver. B.01.01 software and equipped with a reversed-phase Luna C_18_(2) column (Phenomenex, 250 × 21.2 mm, 5 µm, 100 Å). The column was operated at room temperature, the flow rate was maintained at 20 mL/min, using a binary mixture of water–acetonitrile (95:5 *v*/*v*) as eluent A and acetonitrile–water (95:5 *v*/*v*) as eluent B, both acidified with 0.1% formic acid. A volume of 900 µL 50 mg/mL extract was injected and the following gradients were used: standard gradient to obtain Fr.1: 0 min, 0% B; 30 min, 100% B; 35 min, 100% B; 36 min, 0% B and 5 min of equilibration; gradient VI to isolate active compounds of *F. racemosa* EtOAc extract: 0 min, 40% B; 50 min, 75% B; 51 min, 100% B; 56 min, 100% B; 57 min, 40% B; and 5 min of equilibration.

### 3.7. HPLC-HRMS-SPE-NMR Analysis

HPLC-HRMS-SPE-NMR analysis was performed on a platform consisting of an Agilent 1260 chromatograph (Santa Clara, CA, USA), a Bruker micrOTOF-Q II mass spectrometer (Bruker Daltonik, Bremen, Germany), a Knauer Smartline 120 pump (Knauer, Berlin, Germany), a Prospekt-2 SPE unit (Spark Holland, Emmen, The Netherlands), a Gilson 215 liquid handler (Gilson, Middleton, WI, USA), and a Bruker Avance III 600 MHz NMR spectrometer. Agilent 1260 system consists of an auto sampler, a quaternary pump with integrated degasser, a thermostatted column compartment, and a photodiode-array detector. Separation was performed with column, temperature, solvent composition and flow rate as described above, using the following elution profile (gradient VI): 0 min, 40% B; 50 min, 75% B; 51 min, 100% B; 56 min, 100% B; 57 min, 40% B; and 5 min of equilibration. The column eluate was connected to a T-piece splitter to direct 1% of the flow to a micrOTOF-Q II mass spectrometer, equipped with an electrospray ionization (ESI) interface. Mass spectra were acquired in positive ion mode, using drying temperature of 200 °C, capillary voltage of 4100 V, nebulizer pressure of 2.0 bar, and drying gas flow of 7 L/min. The rest of the HPLC eluate (99%) was directed to the photo-diode array (PDA) detector and subsequently diluted with 1.0 mL/min of water by a Knauer Smartline 120 pump, prior to trapping on 10 × 2 mm i.d. Resin GP (general purpose, 5–15 µm, spherical shape, polydivinyl-benzene solid phase) SPE cartridges from Spark Holland. Before trapping, the cartridges were preconditioned with 500 µL of acetonitrile and subsequently equilibrated with 500 µL of water. Selected peaks were trapped cumulatively after 10 consecutive separations using UV absorption-thresholds to trigger trapping. The loaded SPE cartridges were dried with a stream of nitrogen gas for 45 min, and subsequently eluted with 30 µL deuterated acetonitrile-*d*_3_ into 1.7 mm NMR tubes (Bruker Biospin, Germany) using a Gilson 215 liquid handler. The tubes were filled with 30 µL of eluted sample and sealed with plastic balls. HPLC separations, mass spectrometric measurements, and analyte trapping on SPE cartridges were controlled using Hystar ver. 3.2 software (Bruker Daltonik, Bremen, Germany), whereas cartridge elution was controlled by Prep Gilson ST ver. 1.2 software (Bruker Biospin, Karlsruhe, Germany).

### 3.8. NMR Experiments 

All NMR spectra were recorded at 300 K either in acetonitrile-*d*_3_ (for material analyzed in the HPLC-HRMS-SPE-NMR mode) or in acetone-*d*_6_ (for material isolated on preparative scale). NMR experiments were performed using either a Bruker Avance III 600 MHz NMR spectrometer (^1^H operating frequency 600.13 MHz) equipped with a Bruker SampleJet sample changer and a cryogenically cooled gradient inverse triple-resonance 1.7 mm TCI probe-head (Bruker Biospin, Rheinstetten, Germany) or a 600 MHz Bruker Avance III HD spectrometer equipped with a cryogenically cooled 5 mm DCH probe optimized for ^13^C and ^1^H observation. Bruker standard pulse sequences were used throughout this study. Icon NMR version 4.2 (Bruker Biospin) was used for controlling automated acquisition of NMR data (temperature equilibration to 300 K, optimization of lock parameters, gradient shimming, and setting of receiver gain). One-dimensional ^1^H- and ^13^C-NMR spectra were acquired with 30°-pulses, 3.66 s inter-pulse intervals and 64k data points. NMR data acquisition and processing was performed using Topspin version 3.5 (Bruker Biospin).

### 3.9. Kinetics of PTP1B Inhibition 

Enzyme kinetics of isolated PTP1B inhibitors was measured in triplicate using the standard assay conditions described above. Reactions were carried out with increasing concentrations of *p*NPP (0.125, 0.25, 0.5, 1.0, 2.0 and 4.0 mM) as substrate in the absence and presence of test samples (4 concentrations). The mode of inhibition was determined graphically from the Lineweaver-Burk plots. Kinetic parameters were calculated by fitting the data to the Michaelis–Menten equation using non-linear regression analysis (GraphPad Prism 7.0, GraphPad Software Inc., La Jolla, CA, USA).

## 4. Conclusions

In conclusion, PTP1B inhibitory activity of 40 extracts from 18 Vietnamese medicinal plants was measured in vitro. Our study showed that 21 extracts significantly suppressed PTP1B enzyme. The study also demonstrated that *F. racemosa* can be used as a natural source of PTP1B inhibitors. From this plant, PTP1B inhibitory activity has already been reported for three known compounds and PTP1B inhibitory activity has been reported for the first time for one known compound. This is also the first report on the kinetics of PTP1B inhibition of these compounds. The use of the high-resolution PTP1B inhibition profile combined with HPLC-HRMS-SPE-NMR has proved to be a valuable targeting tool for analysis of a crude plant extract.

## Figures and Tables

**Table 1:** IC_50_ values (µg/mL) of extracts showing more than 80% PTP1B inhibitory activity at a concentration of 100 µg/mL.

				IC_50_ (µg/mL)	
Scientific Name	Family	Part Used	Voucher No.	EtOAc Extract	*n*-BuOH Extract
*Nepenthes mirabilis* (Lour.) *Druce*	Nepenthaceae	Whole plant	VN-01	1.4 ± 0.05	0.4 ± 0.1
*Ludwigia octovalvis* (Jacq.) P.H. Raven	Onagraceae	Aerial part	VN-02	16.9 ± 3.2	3.3 ± 0.3
*Phyllanthus amarus Schumach*. & Thonn.	Phyllanthaceae	Whole plant	VN-03	74.4 ± 3.9	n.t.
*Phyllanthus urinaria* L.	Phyllanthaceae	Whole plant	VN-04	14.0 ± 0.8	10.8 ± 0.9
*Phyllanthus reticulatus* Poir.	Phyllanthaceae	Stem, leaf	VN-05	n.t.	n.t.
*Scoparia dulcis* L.	Scrophulariaceae	Whole plant	VN-06	n.t.	n.t.
*Mirabilis jalapa* L.	Nyctaginaceae	Root	VN-07	n.t.	n.t.
		Arial part		n.t.	n.t.
*Cassia fistula* L.	Leguminosae	Leaf	VN-08	24.1 ± 8.4	n.t.
*Lagerstroemia speciosa* (L.) Pers.	Lythraceae	Leaf	VN-09	n.t.	19.6 ± 5.1
*Syzygium cumini* (L.) Skeels	Myrtaceae	Fruit	VN-10	27.5 ± 7.8	n.t.
*Euphorbia hirta* L.	Euphorbiaceae	Whole plant	VN-11	29.2 ± 6.2	38.3 ± 1.9
*Rhizophora mucronata* Lam.	Rhizophoraceae	Bark	VN-12	17.2 ± 1.2	1.8 ± 0.4
*Kandelia candel* (L.) Druce	Rhizophoraceae	Bark	VN-14	12.9 ± 2.2	0.02 ± 0.01
*Cardiospermum halicacabum* L.	Sapindaceae	Whole plant	VN-14	n.t.	n.t.
*Pandanus odoratissimus* L.f.	Pandanaceae	Fruit	VN-15	20.8 ± 5.6	40.4 ± 7.9
*Morinda citrifolia* L.	Rubiaceae	Fruit	VN-16	n.t.	n.t.
*Ficus racemosa* L.	Moraceae	Fruit	VN-17	38.3 ± 10.6	3.6 ± 1.4
*Pithecellobium dulce* (Roxb.) Benth.	Leguminosae	Stem	VN-18	26.1 ± 2.5	n.t.
		Leaf		n.t.	n.t.

n.t.: IC_50_ not tested because inhibition was less than 80% at 100 µg/mL. Values are expressed as mean ± standard deviation (*n* = 3).

**Table 2:** IC_50_, *K*_i_ and mode of inhibition for compounds **1**–**4** on PTP1B.

Compound	IC_50_ (µM)	*K*_i_ (µM)	Mode of Inhibition
Isoderrone (**1**)	22.7 ± 1.7	21.3 ± 2.8	Non-competitive
Derrone (**2**)	12.6 ± 1.6	7.9 ± 1.9	Non-competitive
Alpinumisoflavone (**3**)	21.2 ± 3.8	14.3 ± 2.0	Non-competitive
Mucusisoflavone B (**4**)	2.5 ± 0.2	3.0 ± 0.5	Non-competitive
RK682 ^a^	10.4 ± 1.6	n.t.	n.t.

n.t.: not tested. Values are expressed as mean ± standard deviation (*n* = 3). ^a^ Positive control.

## Supplementary Materials

Supplementary material are available online.
