# Supplementary material for: High-Resolution Inhibition Profiling Combined with HPLC-HRMS-SPE-NMR for Identification of PTP1B Inhibitors from Vietnamese Plants
Source: Molecules. 2017 Jul 20;22(7):1228. doi: 10.3390/molecules22071228 (PMC6152321; doi:10.3390/molecules22071228)

Supplementary data for

High-Resolution Inhibition Profiling Combined with HPLC-HRMS-SPE-NMR for Identification of PTP1B Inhibitors from Vietnamese Plants Used for the Treatment of Type 2 Diabetes

Binh T. D. Trinh, Anna K. Jäger* and Dan Staerk

Department of Drug Design and Pharmacology, Faculty of Health and Medical Sciences, University of Copenhagen, Universitetsparken 2, DK-2100 Copenhagen, Denmark

* Correspondence: anna.jager@sund.ku.dk ; Tel.: +45-35336339

Figure S1 – Dose-response curves of active EtOAc extracts in PTP1B assay. Each point represents the average of triplicate measurements.

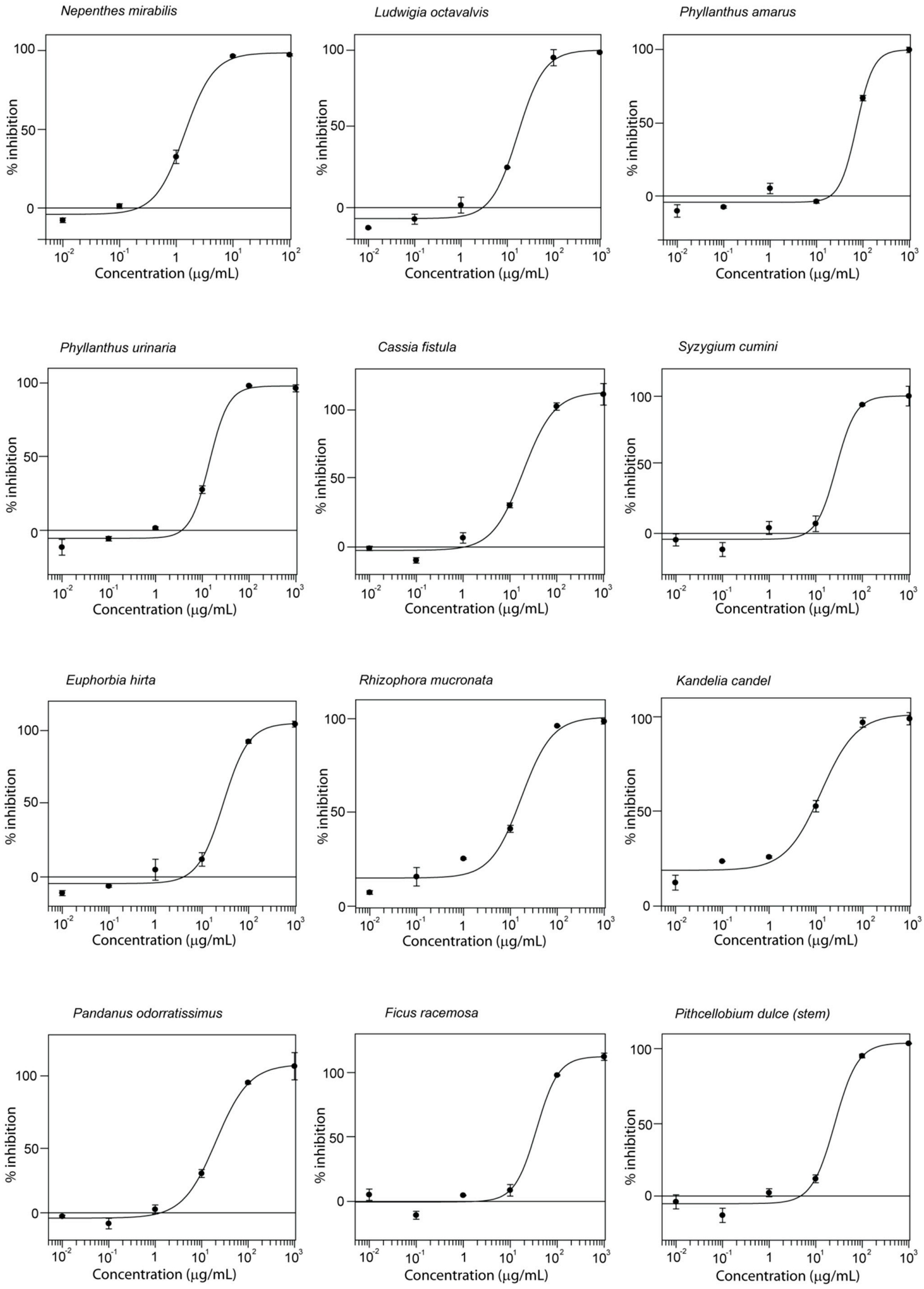

Figure S2 – Dose-response curves of active *n*-BuOH extracts in PTP1B assay. Each point represents the average of triplicate measurements.

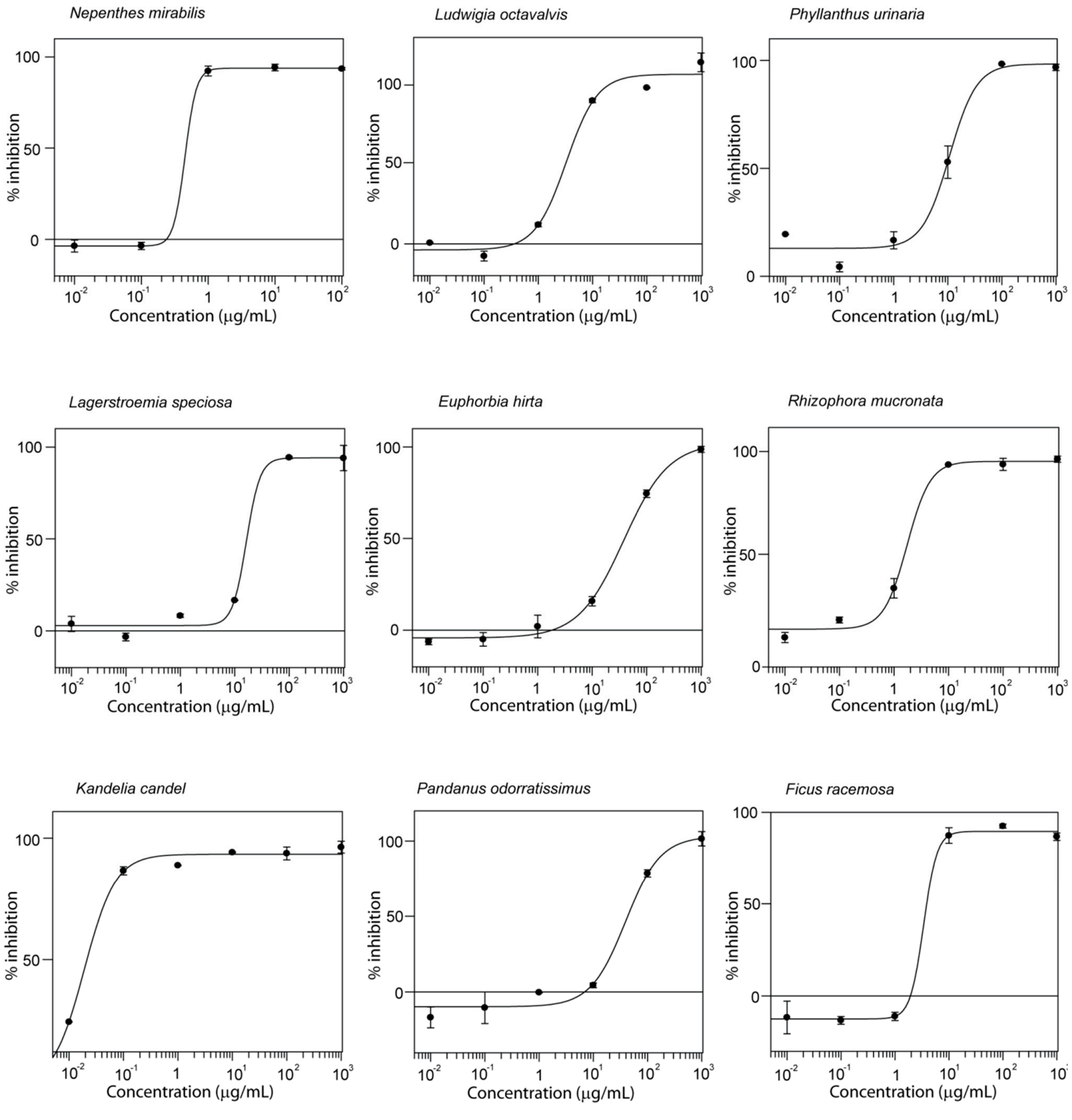

Figure S3 – UV chromatograms monitored at 254 nm of active EtOAc extracts in PTP1B assay.

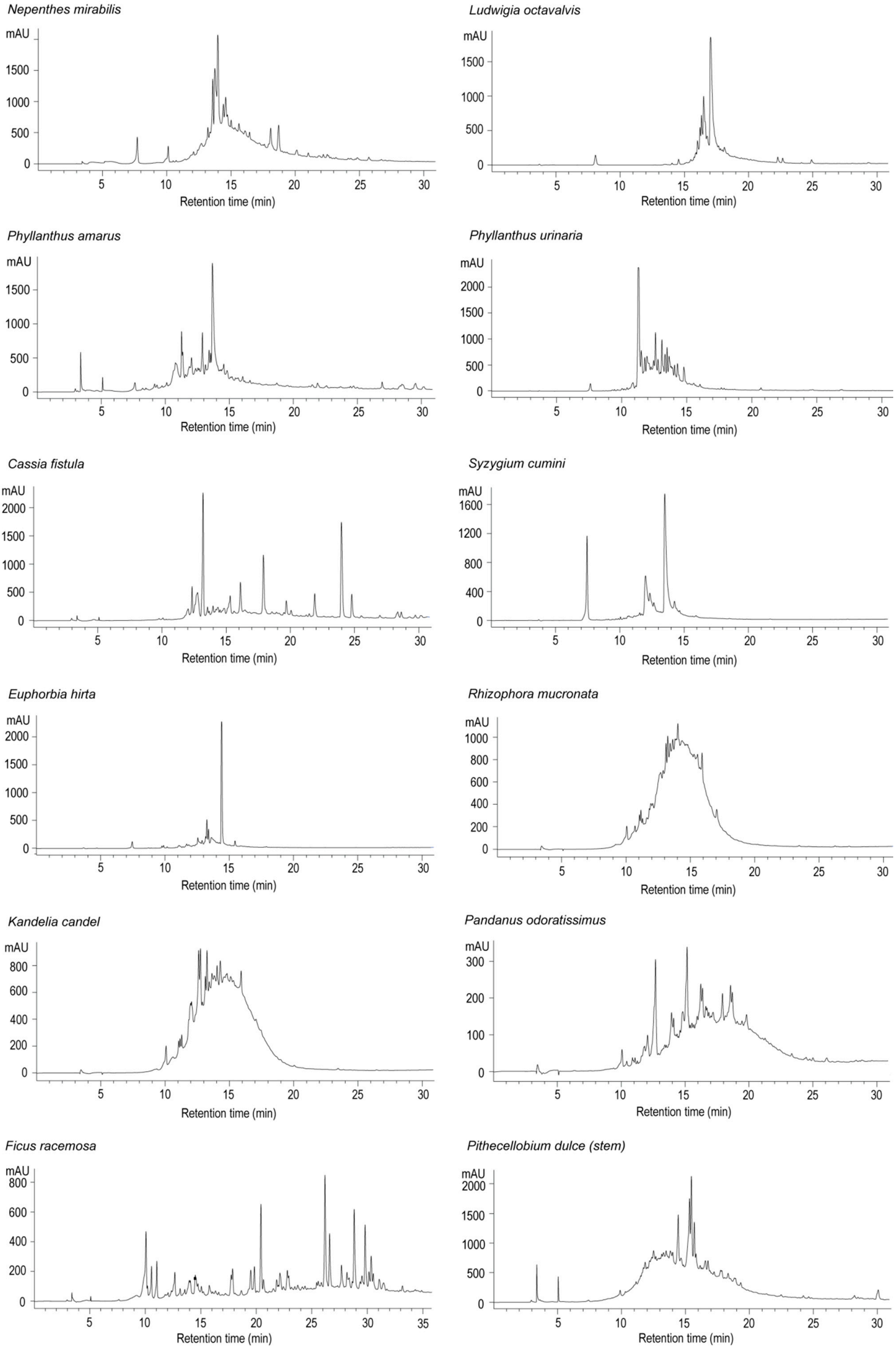

Figure S4 – UV chromatograms monitored at 254 nm of active *n*-BuOH extracts in PTP1B assay.

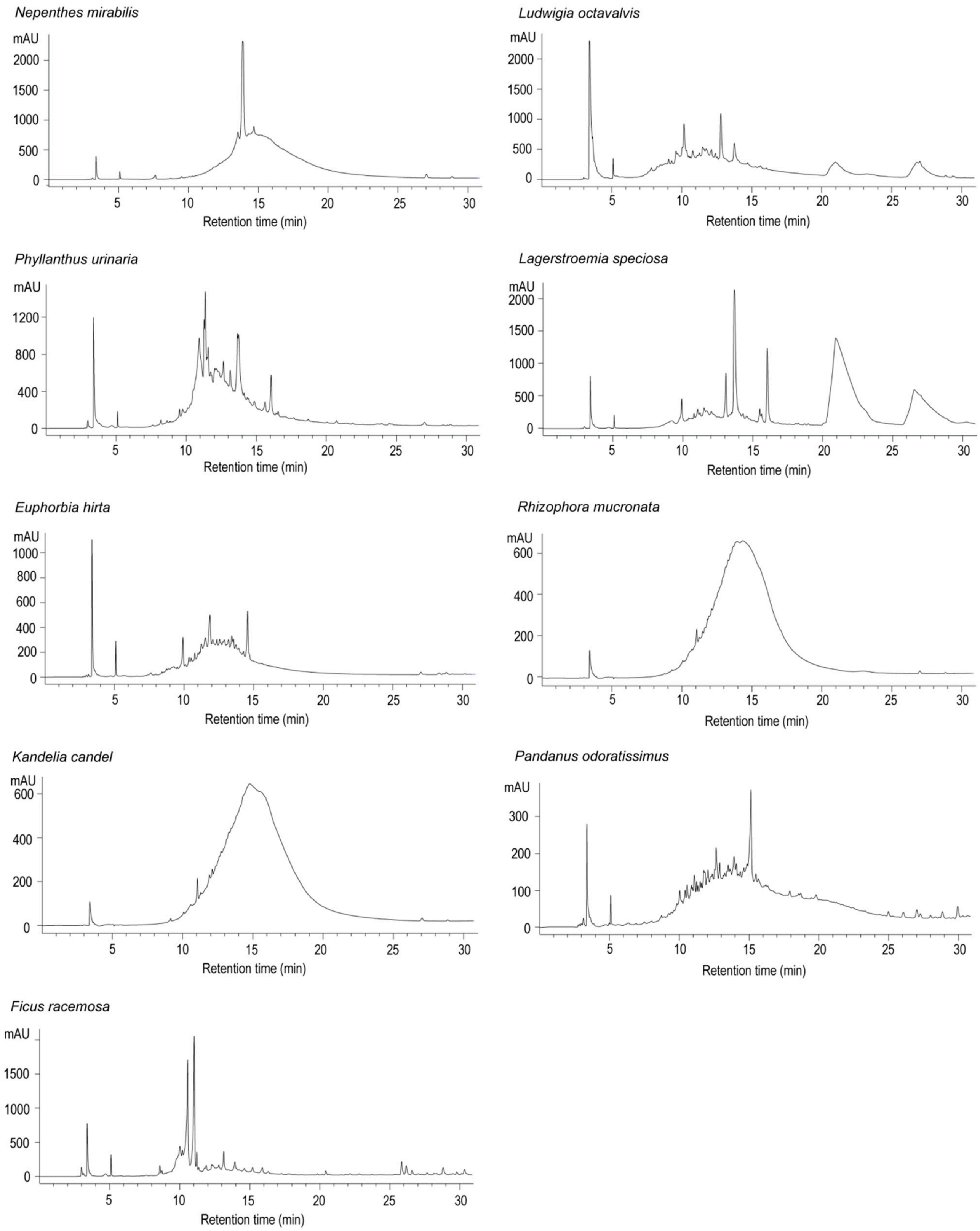

Figure S5 – HPLC trace at 254 nm and high-resolution PTP1B inhibition profile of selected active extracts.

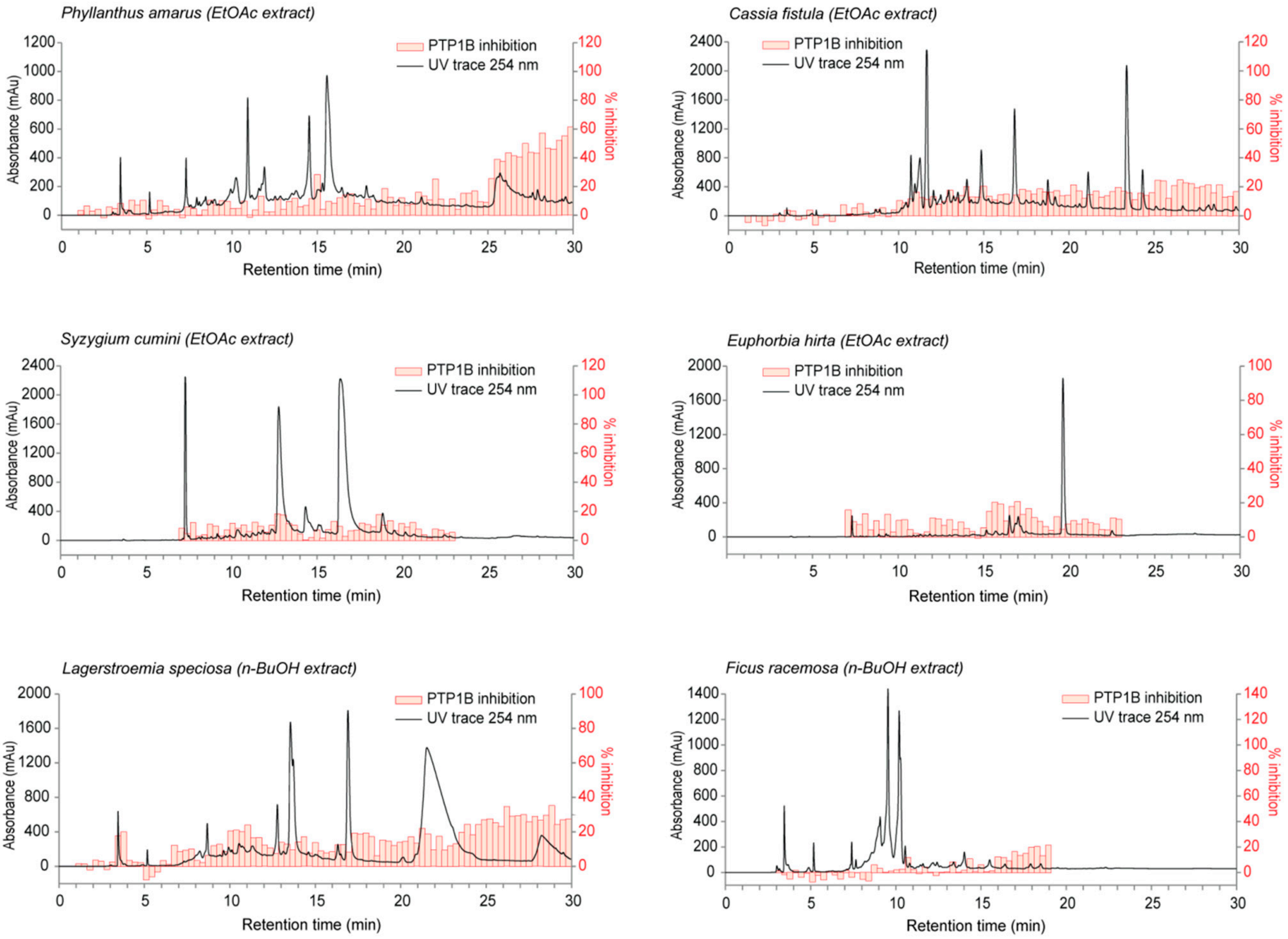

Supplement: Supplementary file 1 [file molecules-22-01228-s001.pdf]
